# Enhancing reliability and automation of LLM-based structural analysis using a hybrid multi-agent pipeline

**DOI:** 10.1038/s41598-026-50127-8

**Published:** 2026-04-27

**Authors:** Seokjae Heo

**Affiliations:** School of Architecture, Dankook University, Gyeonggi-do 16890 Yongin, South Korea

**Keywords:** Large language models, Multi-agent systems, Model context protocol, Structural analysis, Engineering, Mathematics and computing

## Abstract

Large language models (LLMs) can assist engineering workflows, but their direct application in structural analysis is fundamentally limited by numerical inconsistencies, prompt sensitivity, and context degradation. To address these bottlenecks, a verification-and-refinement driven multi-agent framework is proposed. The five-stage pipeline uses an explicit verify–correct loop to improve engineering consistency and incorporates a Model Context Protocol (MCP) hybrid path. This MCP architecture routes highly complex numerical structural calculations to a deterministic external solver under predefined trigger conditions. The methodology was evaluated on two cases: (A) a benchmark frame collapse-analysis problem resolved purely within the LLM verify–correct loop, and (B) an eight-story steel moment-resisting frame where trigger-based routing invoked MATLAB for high-DOF second-order analysis. Across the evaluations, the iterative pipeline increased verification pass rates, with the largest gain occurring during the first verify–correct iteration. Staged prompts and structured JSON/Markdown handoffs also limited context inflation. In Case B, the MCP-delegated numerical solve returned reports that satisfied drift and equilibrium requirements. These results suggest that an iterative verification pipeline combined with policy-guided external-solver delegation can improve reliability in complex structural engineering problems.

## Introduction

Large language models (LLMs) have driven rapid innovation in natural language processing and are increasingly being explored as engineering assistants for code interpretation, design-code consultation, preliminary design exploration, and technical reporting^1–3^. However, their direct application to structural analysis remains challenging. First, existing LLMs can produce numerical mistakes or hallucinated outputs when multi-step calculations must remain consistent with physical laws^4,5^. In structural-analysis settings, such errors may appear as inconsistent load combinations, equilibrium violations, or unrealistic responses caused by incorrect unit handling. Second, structural analysis demands reliability and reproducibility, yet LLM outputs may vary even for the same problem because prompt phrasing, ordering of information, or accumulated context can change the generated solution path^5^. Third, many structural-analysis tasks contain extensive contextual information, including drawings, boundary conditions, load cases, and design criteria, and long-context degradation can make it harder for an LLM to preserve early assumptions throughout a lengthy solution chain^6^. These issues are consequential because support conditions, member properties, code limits, and target outputs must remain consistent from problem definition through final reporting.

To address these limitations, a five-stage multi-agent pipeline is introduced for structural-analysis tasks. The framework draws on the way human engineers decompose complex problems into substeps and iteratively review intermediate results before finalizing an answer. The proposed workflow therefore separates the task into Solver, Self-Improvement, Verifier, Correction, and Synthesis stages, allowing each stage to inspect or refine the previous artifact before the final report is assembled. This design is informed by prior work on self-consistency, tree-structured reasoning, verbal self-correction, and multi-agent verification in LLM systems^7–12^. Here, these ideas are adapted to structural analysis by requiring traceable intermediate outputs and explicit engineering checks rather than relying on a single monolithic response.

Unlike general reasoning benchmarks, structural analysis also requires case-specific physical validation. Global equilibrium, admissible collapse interpretation, deformation compatibility, drift or code checks, and consistency between model assumptions and reported results remain necessary even when an LLM produces a fluent explanation^13–16^. The Verifier stage was therefore designed as an engineering audit layer that evaluates intermediate or returned results against first-principles requirements, and the verify—correct loop uses those findings to improve reliability before synthesis.

**Fig. 1:**
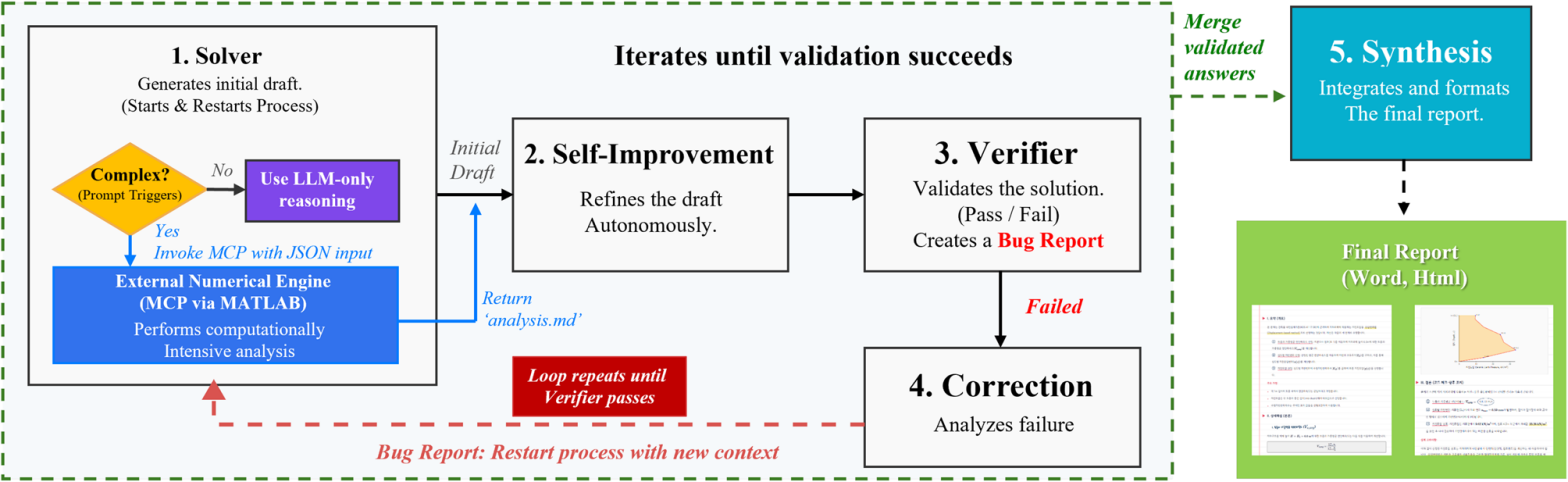
Overview of the 5-stage multi-agent pipeline architecture.

An MCP (Model Context Protocol) hybrid architecture is also introduced for numerically intensive subtasks. While LLMs are useful for problem interpretation, decomposition, and report synthesis, they are inefficient for sparse matrix solving, eigenvalue calculations, and nonlinear iterative analysis at larger scales. To address that gap, the MCP path routes selected numerical subproblems to MATLAB under a predefined trigger policy rather than open-ended self-judgment. When one of the trigger conditions is met, the Solver packages geometry, material, boundary-condition, loading, and analysis-option data into a schema-constrained JSON handoff, and the returned results are provided as a Markdown analysis report for downstream checking. The LLM-side Verifier then examines integration-level engineering consistency before the final report is synthesized. This design combines structured LLM orchestration with deterministic numerical backends while limiting uncontrolled context growth. Figure 1 summarizes the overall workflow.

This paper is organized as follows. First, the background section reviews prior work on self-correction, multi-agent reasoning, structured outputs, tool use, and structural-engineering applications of LLMs. The methodology section then defines the five-stage pipeline, the MCP trigger policy, and the evaluation protocol. The results section evaluates the framework through two case studies: (Case A) a benchmark frame collapse-analysis problem solved with the LLM-only pipeline, and (Case B) an eight-story steel frame analyzed under the MCP-hybrid workflow. Finally, the discussion and conclusion interpret the findings, limitations, and future directions of the study.

## Background

Research on systematizing LLM reasoning and introducing verification feedback has developed along several related directions. Recent work has examined the limits of LLM self-correction, showing that improvement is possible but highly dependent on task type, prompt structure, and the quality of feedback delivered to the model^9,11^. Other studies have shown that closed verification-and-refinement pipelines and multi-agent critique setups can improve reliability on difficult reasoning tasks by separating generation from checking and correction^10,12,17^. The five-stage pipeline used here is aligned with that broader line of work: complex tasks tend to be handled more robustly when intermediate outputs are audited, revised, and only then passed forward. This verification-oriented logic is extended to structural-analysis problems, where the output must satisfy engineering constraints rather than only appear linguistically coherent.

Research on structuring LLM outputs and validating response formats is also directly relevant. Constraining responses to structured formats such as JSON can simplify parsing and downstream processing, but models do not always comply with strict format requirements without additional control layers^18^. Recent guardrail approaches therefore use schemas and declarative constraints to check whether outputs satisfy predefined fields, types, and logical conditions before they are accepted^19^. Related orchestration frameworks such as DSPy formalize multi-stage prompt flows and make it easier to define reproducible language-model pipelines with explicit interfaces between stages^20^. Long-context studies add a further motivation for such design choices: when inputs become lengthy, information located away from the prompt boundaries can be used less effectively, which is problematic for engineering tasks that must preserve early assumptions and boundary conditions throughout a full solution chain^6^. These findings motivate the structured handoff and stage-wise context management strategy used in the present framework.

Within structural engineering and architecture, early LLM applications have focused mainly on text-centered tasks such as code consultation, BIM-linked compliance checking, and domain-specific question answering^1–3^. More recent work has started to examine agentic LLM workflows for improving reliability in structural-analysis settings^21^. Even so, the current literature still provides limited evidence for workflows that explicitly combine staged LLM verification, structured tool handoff, and deterministic numerical backends on problems that require physically admissible structural-analysis outputs. The gap addressed here is the use of LLM reasoning as one part of a larger analysis workflow rather than as a standalone replacement for numerical analysis software.

The engineering-verification side of the problem has its own long history. Numerical and structural-analysis practice has long relied on explicit post-checking criteria, including virtual-work-based reasoning, equilibrium checks, and second-order consistency evaluation^13–15^. In frame and building analysis, physically meaningful acceptance often requires more than a fluent summary of computed results: drift limits, load-path consistency, collapse admissibility, and compatibility of reported reactions and member actions must also be checked. Seismic and serviceability studies likewise emphasize the need to verify whether deformations and response quantities remain within acceptable ranges under the governing assumptions^16^. In the present study, this tradition motivates the Verifier’s engineering checklist, which focuses on equilibrium, collapse-mechanism admissibility, plastic-moment consistency, drift/code checks, and agreement between transmitted models and returned reports.

These prior studies suggest that staged LLM reasoning, structured intermediate artifacts, external tool integration, and explicit engineering verification can be combined productively. The following section introduces the detailed structure and operating principles of the five-stage multi-agent pipeline and the MCP-based hybrid architecture developed from these insights.

## Methods

All agents in the proposed pipeline were implemented with Google’s Gemini 2.5 Pro model. To reduce stochastic variability during the evaluation, the temperature parameter was set to 0.1 and external web search was disabled. This setting was intended to evaluate the effect of the orchestration design itself rather than the effect of retrieval augmentation.

The overall structure of the proposed pipeline is illustrated in Fig. 1. After receiving the problem definition, the workflow executes five predefined agent stages. Each stage receives only the information needed for its role and returns a structured artifact for the next stage, which limits uncontrolled context growth and leaves a traceable verification record.

### Research questions and evaluation metrics

Two research questions guided the evaluation. *RQ1* asked whether the five-stage verify–correct pipeline improves verification pass rates and output stability relative to a single-thread comparison workflow. *RQ2* asked whether predefined MCP trigger conditions improve handling of higher-complexity structural-analysis tasks by routing selected numerical subproblems to an external deterministic solver.

Primary quantitative metrics were (i) final verification pass rate, (ii) Stage-3 Verifier pass rate, (iii) run-to-run dispersion across repeated runs, and (iv) the Context Inflation Ratio (CIR). Case-specific engineering checks included equilibrium, collapse-mechanism admissibility, plastic-moment-limit consistency, and interstory drift compliance.

### Experimental setting and repeated-run protocol

The proof-of-concept case studies in “Case A: benchmark frame collapse-analysis problem (LLM-only)” and “Case B: 8-story steel moment frame analysis (policy-guided MCP hybrid)” were used to examine task-level behavior of the LLM-only pipeline and the MCP-hybrid workflow. For the repeated-run analyses reported in Figs. 11, 12, 13, the pipeline was evaluated on 45 sessions of the Korean Professional Engineer Structural Engineering examination (1350 problems in total). Each session was processed independently 10 times under the same model setting. The comparison baseline retained the same model family but appended correction history within a single thread, allowing context to grow monolithically rather than resetting by stage.

### Solver agent (stage 1)

**Fig. 2:**
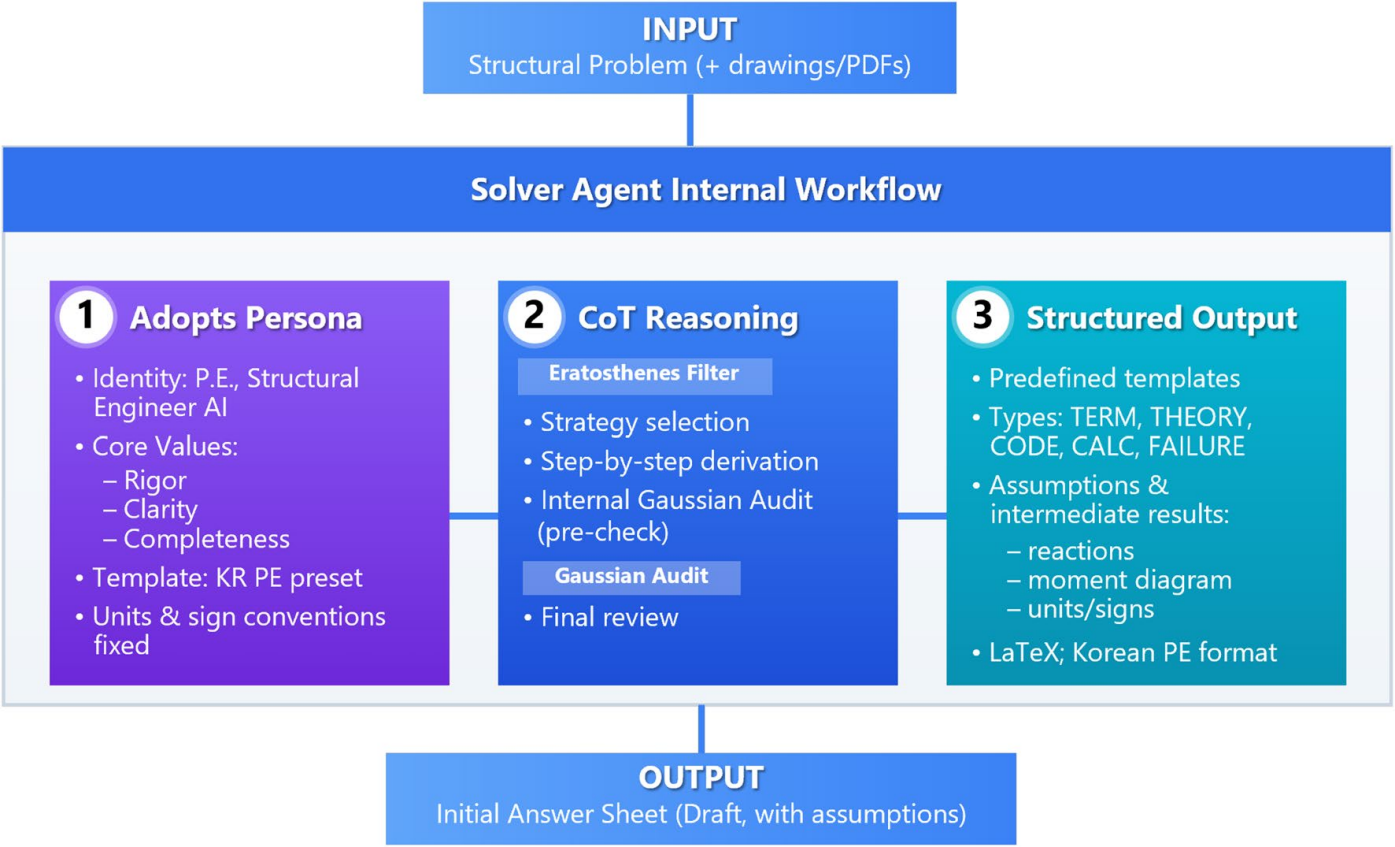
Internal workflow and structured output format of the Solver agent.

The Solver receives the problem statement, relevant configuration data, and the current routing policy. Its task is to produce the first structured solution draft, including the selected analysis strategy, explicit assumptions, intermediate calculations, and a report candidate for downstream review. When a problem satisfies the MCP trigger policy, the Solver also prepares the JSON handoff package for the external solver instead of continuing all numerical work internally. The stage terminates after a traceable draft or routing package has been produced, as illustrated in Fig. 2.

### Self-improvement agent (stage 2)

**Fig. 3:**
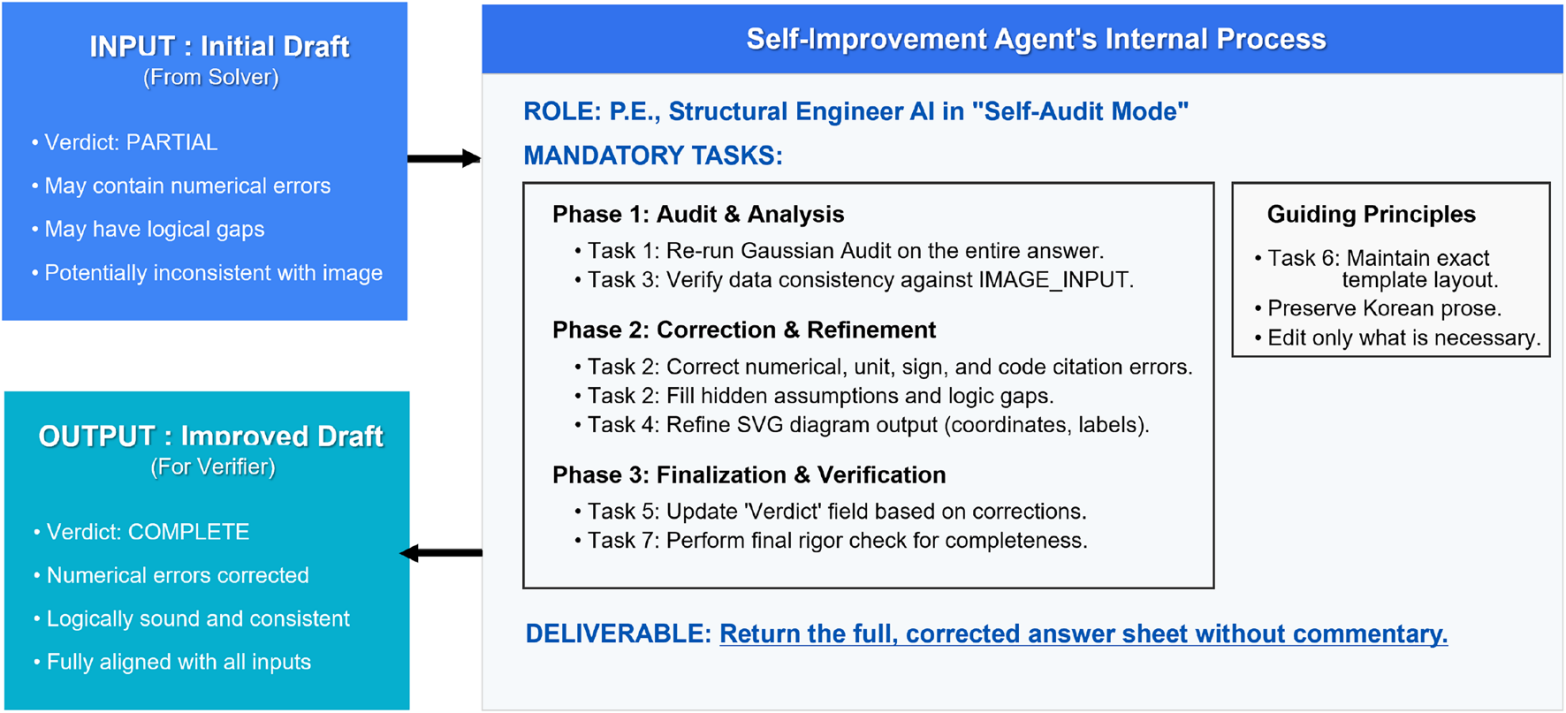
Internal audit and improvement process of the Self-Improvement agent.

The Self-Improvement agent audits the Solver draft from an independent perspective. Its role is to identify missing assumptions, ambiguous sign conventions, incomplete local reasoning, or report elements that are not yet ready for formal verification. The output is a revised draft or annotated returned-report package that is more explicit and internally consistent than the Stage-1 input, as shown in Fig. 3.

### Verifier agent (stage 3)

**Fig. 4:**
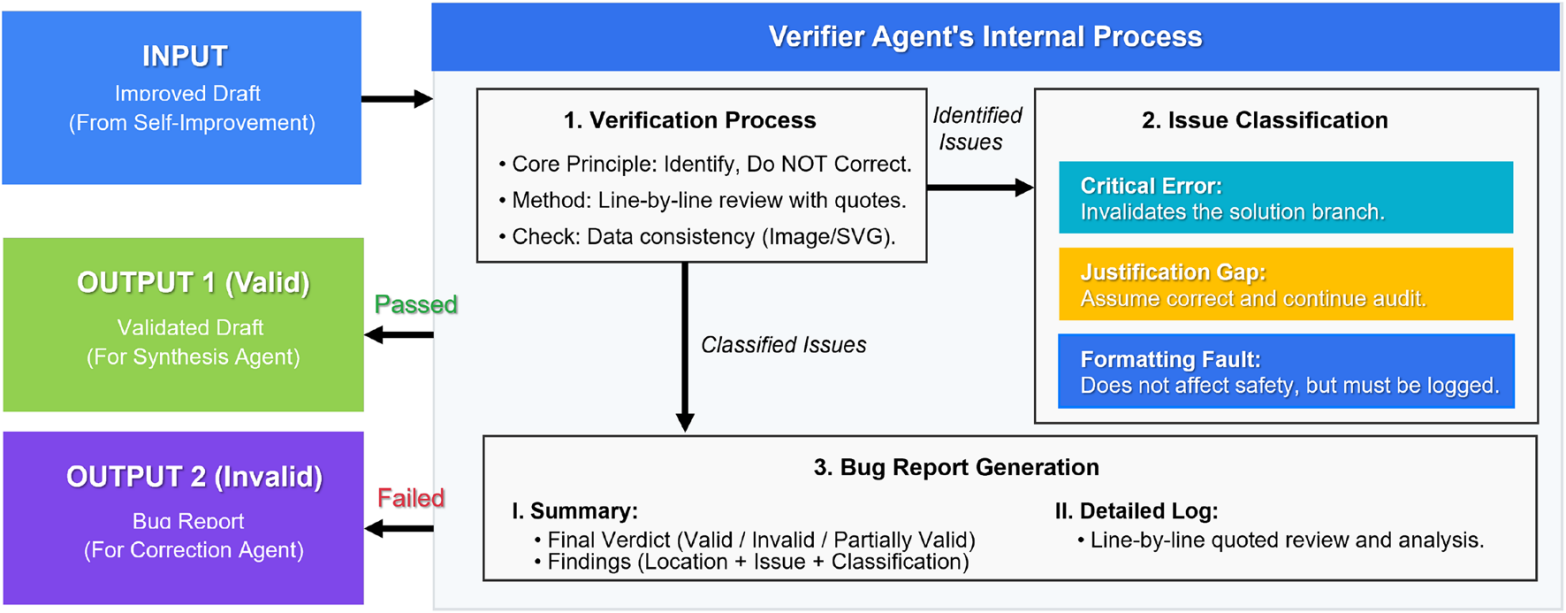
Verification process and error classification system of the Verifier agent.

The Verifier performs an explicit engineering audit of the revised draft. Depending on the case, the checklist includes global equilibrium, unit consistency, admissibility of the proposed collapse mechanism, plastic-moment-limit consistency, drift/code checks, and completeness of the transmitted model description. The Verifier returns a structured bug report with severity labels (Critical, Gap, or Format) together with a final status of Pass or Fail, as shown in Fig. 4.

### Correction agent (stage 4)

**Fig. 5:**
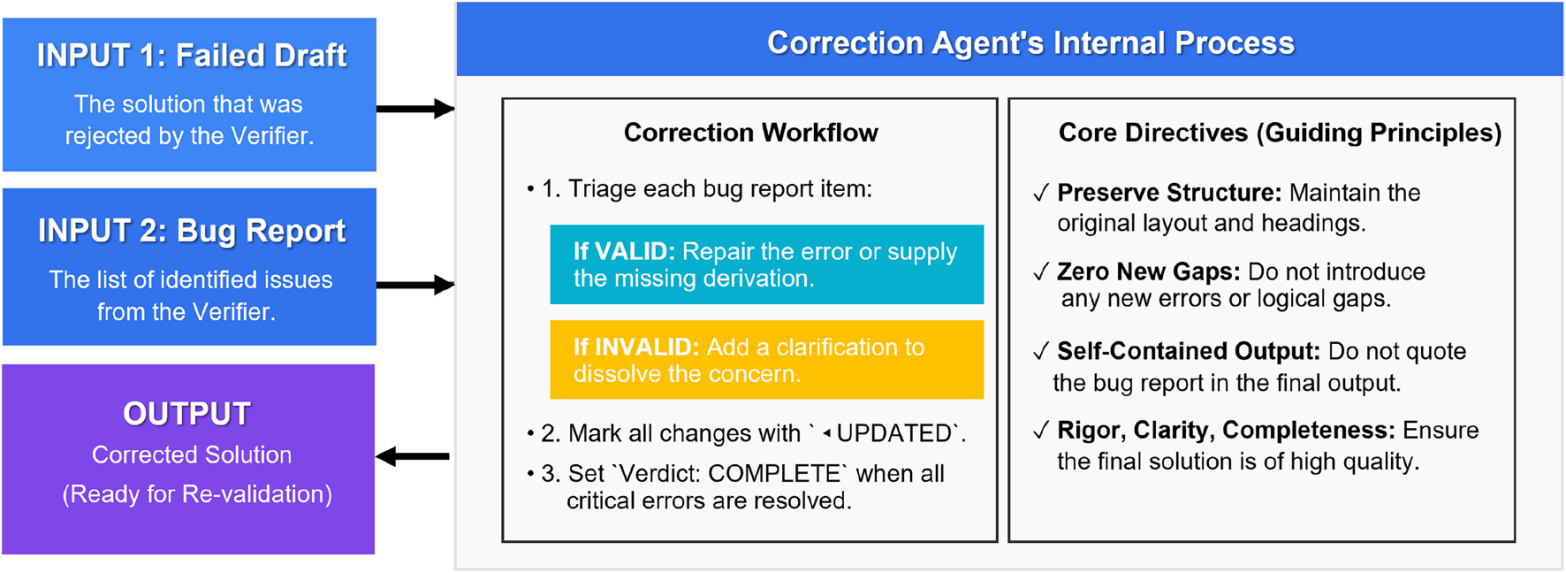
Bug report-based correction workflow of the Correction agent.

The Correction agent consumes the bug report and updates only the flagged portions of the draft. It preserves already validated quantities whenever possible, records the implemented fixes, and resubmits the revised result for re-verification. The stage terminates when all reported issues have been addressed or when no further progress can be made within the current iteration budget, as shown in Fig. 5.

### Synthesis agent (stage 5)

**Fig. 6:**
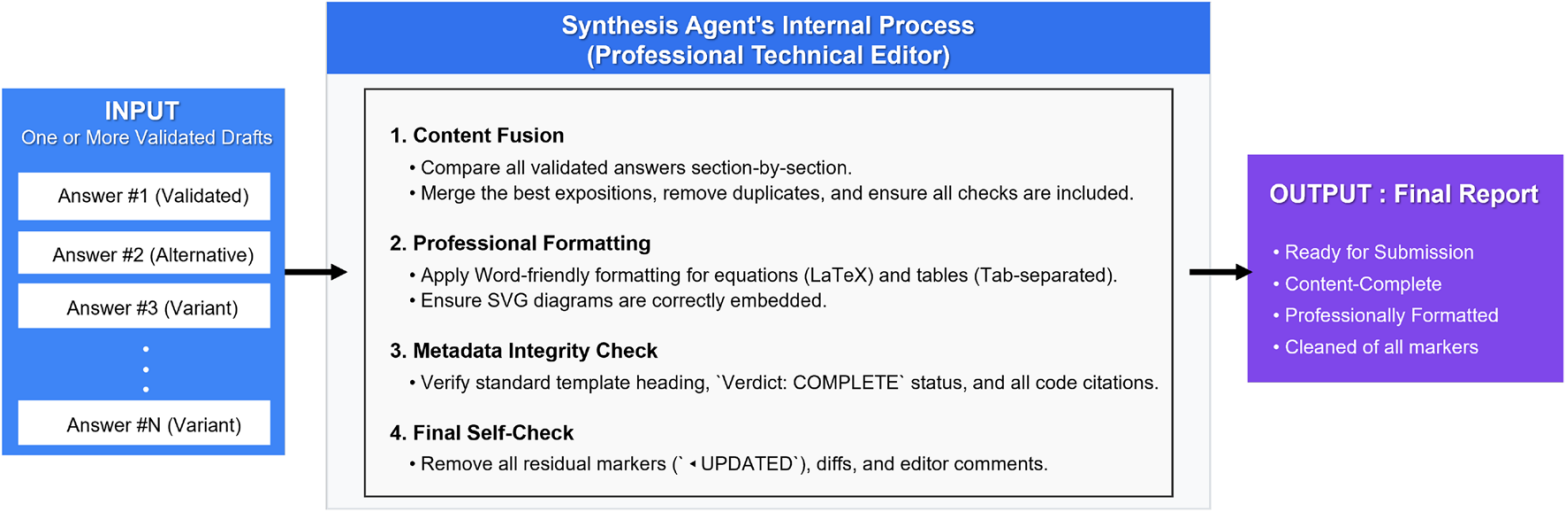
Multiple solution integration and final report generation procedure of the Synthesis agent.

The Synthesis stage aggregates only candidate solutions whose latest Verifier status is Pass. It does not perform new numerical calculations, averaging, or interpolation. Instead, it selects, compresses, and narratively integrates already verified content into a submission-ready report. Any numerical discrepancy discovered at this stage is routed back to the Correction–Verifier loop rather than resolved heuristically inside Synthesis. This preserves the division of responsibility between content integration and numerical verification, as shown in Fig. 6.

### MCP-based hybrid approach

**Fig. 7:**
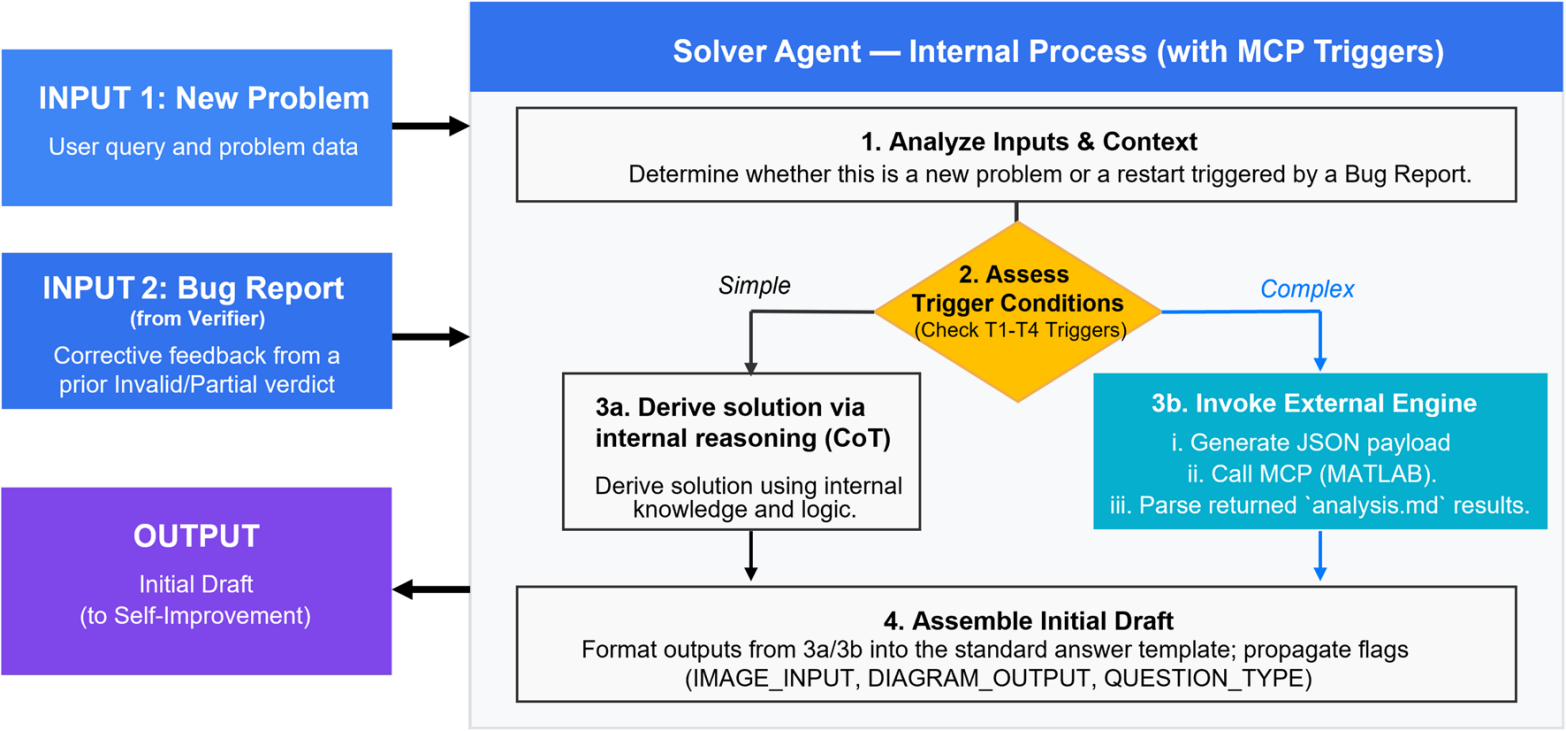
Conditional calling mechanism for external analysis engine using MCP.

To address higher-complexity structural-analysis problems, this study adds an MCP (Model Context Protocol) hybrid architecture to the five-stage pipeline. The LLM remains the orchestrating layer, but routing to the external solver follows a predefined trigger policy rather than open-ended ad hoc choice. Figure 7 illustrates the schematic of this MCP-based hybrid execution pipeline. When the Solver encounters any of the trigger conditions defined below, the relevant numerical subproblem is routed to MATLAB.

**Table 1:** Trigger definition & thresholds.

ID	Condition	Symbol/rule	Notes
T1	High DOF	DOF > 100	Case B: DOF = 108
T2	Nonlinearity	P-$\Delta$ or material NL	Iteration limit $k_{\max }$ (configurable in external solver)
T3	Eigen/buckling	eigen solve flagged	Number of modes *m* (specified by analysis request)
T4	Token-intensive loop	estimated tokens > $\tau$	Element matrix assembly, etc

Table 1 presents the external engine calling trigger conditions set in this study, with details for T1–T4 as follows:

- T1 (large DOF): when the degrees of freedom (DOF) or equation scale to be analyzed is excessively large for direct LLM solution. Applicable when large sparse solving exceeding user-specified DOF is required.
- T2 (nonlinear effects): cases requiring iterative analysis such as P-$\Delta$ effects or material nonlinearity. For example, problem types where accuracy cannot be guaranteed with a single linear solution, such as second-order effects or plastic behavior analysis of high-rise frames.
- T3 (eigenvalue problems): cases requiring eigenvalue calculations such as modal analysis or buckling analysis. Determining natural vibration periods, mode shapes, or critical buckling loads of structures reduces to characteristic matrix eigenvalue problems, requiring specialized numerical analysis.
- T4 (token-intensive iterative tasks): token-intensive tasks requiring calculation through multiple loops in LLM prompts. For instance, assembling hundreds of element stiffness matrices or performing floor-by-floor load aggregation individually causes rapid increases in LLM token consumption, making it inefficient.

When any of these conditions are met, the pipeline packages the problem into input JSON for transmission to the external engine. The JSON includes geometric configuration, material properties, boundary conditions, load definitions, analysis options, and any verified intermediate information that is useful for the downstream solve. Schema-constrained generation is used to reduce parsing errors at the tool boundary.

The external engine (MATLAB) performs the structural analysis and returns a Markdown-format report (analysis.md) containing displacements, reactions, member-force summaries, and analysis options. In this hybrid path, the LLM-side Verifier does not attempt to re-derive every matrix operation performed inside MATLAB. Instead, it checks integration-level engineering consistency: whether the returned report matches the transmitted model, whether equilibrium and drift/code checks are satisfied, and whether the report is complete enough for downstream synthesis. If inconsistencies are found, the pipeline revises the handoff or analysis settings and reruns the external solve.

## Results

### Case A: benchmark frame collapse-analysis problem (LLM-only)

**Fig. 8:**
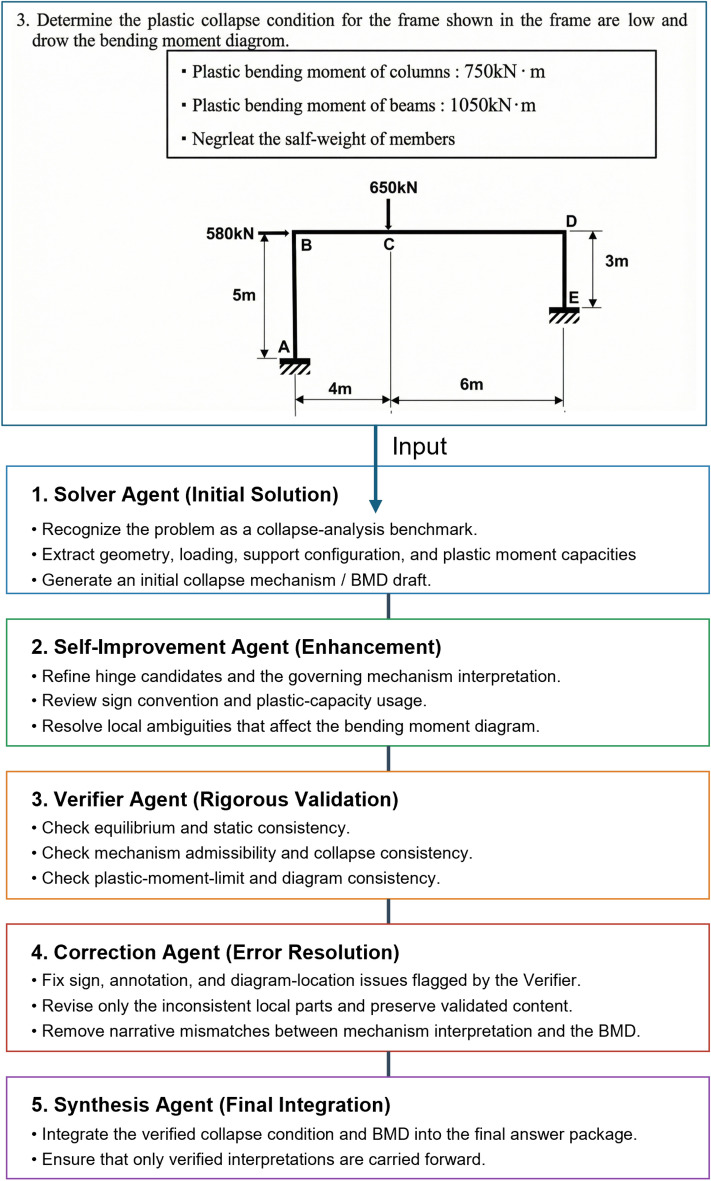
Case study A: application procedure of the 5-stage pipeline for a benchmark frame collapse-analysis problem. The upper panel schematically restates a benchmark item from the public Q-Net past-exam archive for the Korean Professional Engineer Structural Engineering examination, whereas the lower panel summarizes the author-defined pipeline workflow used in this study.

Case A used the benchmark frame statement shown in the upper panel of Fig. 8. That upper panel schematically restates a publicly available item from the Q-Net past-exam archive for the Korean Professional Engineer Structural Engineering examination, and the geometry, supports, loads, and plastic moment capacities used in the analysis were taken from that source. The target outputs were the governing collapse condition and the corresponding bending moment diagram.

The Solver produced an initial collapse-mechanism and bending-moment-diagram draft from the benchmark statement. The Self-Improvement stage then refined hinge candidates, sign conventions, and plastic-capacity assignments that were incompletely stated in the initial draft. The Verifier checked global equilibrium, admissibility of the proposed mechanism, plastic-moment-limit consistency, and local reporting consistency. The Correction stage addressed flagged sign or annotation mismatches, and the Synthesis stage consolidated only the verified interpretation into the final answer package.

In this configuration, the closed verification loop improved the internal coherence of the benchmark solution without changing the problem definition shown in Fig. 8. The final package satisfied the case-specific verification checklist and produced a consistent collapse interpretation and bending moment diagram.

Table 2 summarizes the pipeline operations and key results for Case A by stage. The emphasis is on maintaining consistency between the benchmark problem statement, the interpreted collapse mechanism, and the reported bending moment diagram.

**Table 2:** Case A—summary of key tasks and results for each stage.

Stage	Key tasks	Results
1. Solver	Interpreted the benchmark statement and drafted an initial collapse mechanism and bending moment diagram	Preliminary collapse interpretation produced
2. Self-Improvement	Refined hinge candidates, sign convention, and plastic-capacity usage	Mechanism interpretation clarified and local inconsistencies reduced
3. Verifier	Checked equilibrium, mechanism admissibility, plastic-moment-limit consistency, and reporting coherence	Non-admissible or inconsistent items flagged for revision
4. Correction	Corrected sign, annotation, and diagram mismatches identified in the bug report	Verified diagram and report consistency restored
5. Synthesis	Integrated only the verified collapse condition and bending moment diagram into the final report	Final benchmark answer package prepared

### Case B: 8-story steel moment frame analysis (policy-guided MCP hybrid)

**Fig. 9:**
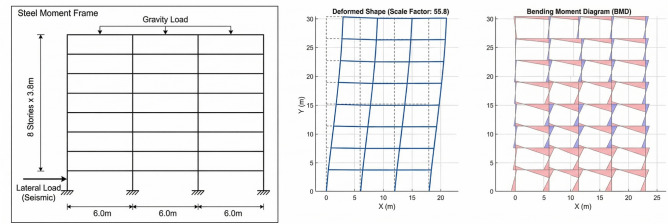
Case study B: 8-story steel moment frame model with representative analysis outputs from the policy-guided MCP workflow.

Case B represents an example of lateral-force analysis for a medium-scale steel building, with the frame schematic shown in Fig. 9. The target structure is an 8-story, 3-bay steel moment-resisting frame with assumed story height of 3.8 m and bay width of 6.0 m. Beam members consist of W24$\times$68 (floors 5–8) and W24$\times$84 (floors 1–4), while column members comprise W14$\times$159 (perimeter) and W14$\times$193 (interior). Equivalent static seismic loads were determined according to a base shear coefficient of 0.12g, with KDS 41 load combination (1.2D + 1.0E + 0.5L) applied for design review. The target outputs were member-force summaries, base reactions, and interstory drift ratios, and the main validation checks were static equilibrium, drift-limit compliance, and consistency between the transmitted model and the returned solver report.

The LLM multi-agent pipeline was applied to this analysis problem, with the MCP hybrid architecture used for the numerically intensive portions. The Solver agent first performed problem parsing and basic preprocessing, including floor-by-floor load setup. During this process, the predefined trigger policy identified that the problem’s degrees of freedom (DOF = 108) exceeded the threshold for T1 and that P-$\Delta$ second-order effects required T2 handling.

Because the trigger conditions were satisfied, the Solver generated a structured JSON handoff containing geometry, member data, loads, boundary conditions, and analysis options for the external MATLAB solver. MATLAB then performed sparse matrix solving and P-$\Delta$ iterative analysis and returned key results, including displacements and member-force distributions, in Markdown report format. In this hybrid path, the Verifier did not attempt to re-prove every deterministic numerical operation inside MATLAB. Instead, it checked integration-level engineering consistency: equilibrium, drift-limit compliance, and agreement between the returned report and the transmitted model definition.

The final analysis results obtained through this process confirmed that the structure satisfied both the allowable interstory drift ratio limit and static equilibrium conditions, with key findings summarized in Table 3.

**Table 3:** Case B—key analysis results of the 8-story steel moment frame.

Item	Value (unit)	Remarks
Total degrees of freedom (DOF)	108	T1 satisfied (> 100)
Maximum interstory drift ratio (occurring floor)	0.00243 (1st floor)	Within limit 0.020
1st floor interior column axial force *N*	7.99 kN	Compression (+)
1st floor interior column shear force *V*	150.66 kN	—
1st floor interior column moment *M* (bottom)	355.31 kN m	Maximum at foundation
1st floor interior column moment *M* (top)	217.18 kN m	2nd floor beam connection
Total base shear	-497.66 kN	Matches sum of foundation horizontal reactions
Overturning moment (foundation level)	11190.50 kN m	Sum of foundation moment reactions

Case B shows that policy-guided routing can support larger structural-analysis tasks within the evaluated workflow. Under the predefined trigger conditions, the external solver handled the selected numerical subproblem while the LLM pipeline maintained engineering checks, report integration, and final synthesis. Rule-based routing was most useful when task complexity exceeded a practical LLM-only regime.

### Effects of the verification loop and iteration gate performance

The following analyses examine two key components of the proposed multi-agent pipeline: (1) the Verification-Correction Loop and (2) the MCP (Model Context Protocol)-based hybrid architecture.

#### Convergence utility of verification-correction loop and gate policy

**Fig. 10:**
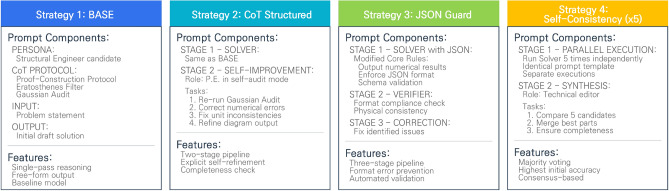
Four prompt engineering strategies evaluated in ablation study.

To quantify the effect of the verification-correction loop on reliability, final pass rate was measured as a function of the maximum allowed iteration count (*k*). To examine how loop effectiveness varied with prompt structure, four prompt families with different levels of output constraint were compared, as shown in Fig. 10:

- Base: baseline model allowing free-form responses without special constraints.
- CoT structured (chain-of-thought): model enhancing reasoning systematicity by guiding logical step-by-step thinking processes.
- JSON guard: model preventing format errors at source by fixing output format to strict JSON schema.
- Self-consistency $\times$ 5: model maximizing initial accuracy by generating five independent responses to the same question and adopting the most consistent result through majority vote.

**Fig. 11:**
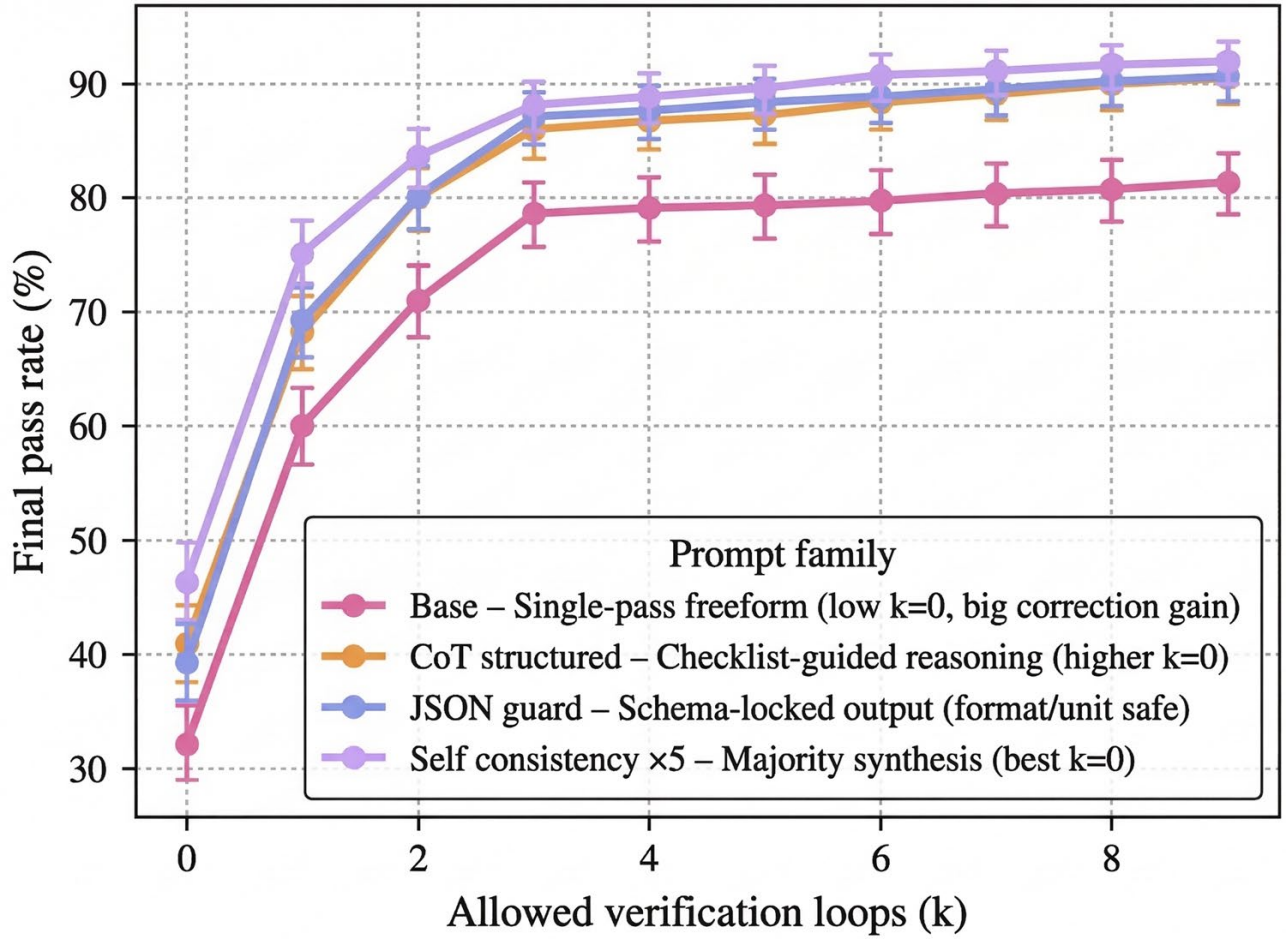
Mean final verification pass rates by allowed verification-correction loop iterations (*k*) across the four prompt families.

This controlled comparison was designed to test whether the proposed loop architecture improves performance across multiple prompt families. Results in Fig. 11 show a consistent pattern across the evaluated prompt designs. The sharpest improvement occurs in the first verification-correction loop ($k=0 \rightarrow 1$). In particular, the least-constrained ‘Base – Single-pass freeform’ design started with the lowest initial pass rate and showed the largest first-loop gain, which is consistent with the interpretation that the Verifier-Correction feedback step removes many obvious or structural errors (Critical/Gap) in early iterations.

Conversely, diminishing marginal returns became apparent after $k \ge 2$. The ‘Self-consistency $\times$5—majority synthesis’ setting achieved the highest initial pass rate at $k=0$ (46.38%), compared with 40.88% for CoT structured, 39.25% for JSON guard, and 32.12% for the Base setting; however, the gap narrowed after iterative verification, reaching 88.12%, 86.00%, 87.12%, and 78.62% by $k=3$, respectively (Supplementary Table S4). This pattern suggests stronger initialization quality, with a smaller remaining advantage after iterative verification, rather than uniformly low loop dependence.

These results favor a verifier-guided stopping rule over a fixed loop count. Most gains were obtained within the first one or two iterations, and later improvements were modest.

#### Context-dependent utility of MCP hybrid architecture

**Fig. 12:**
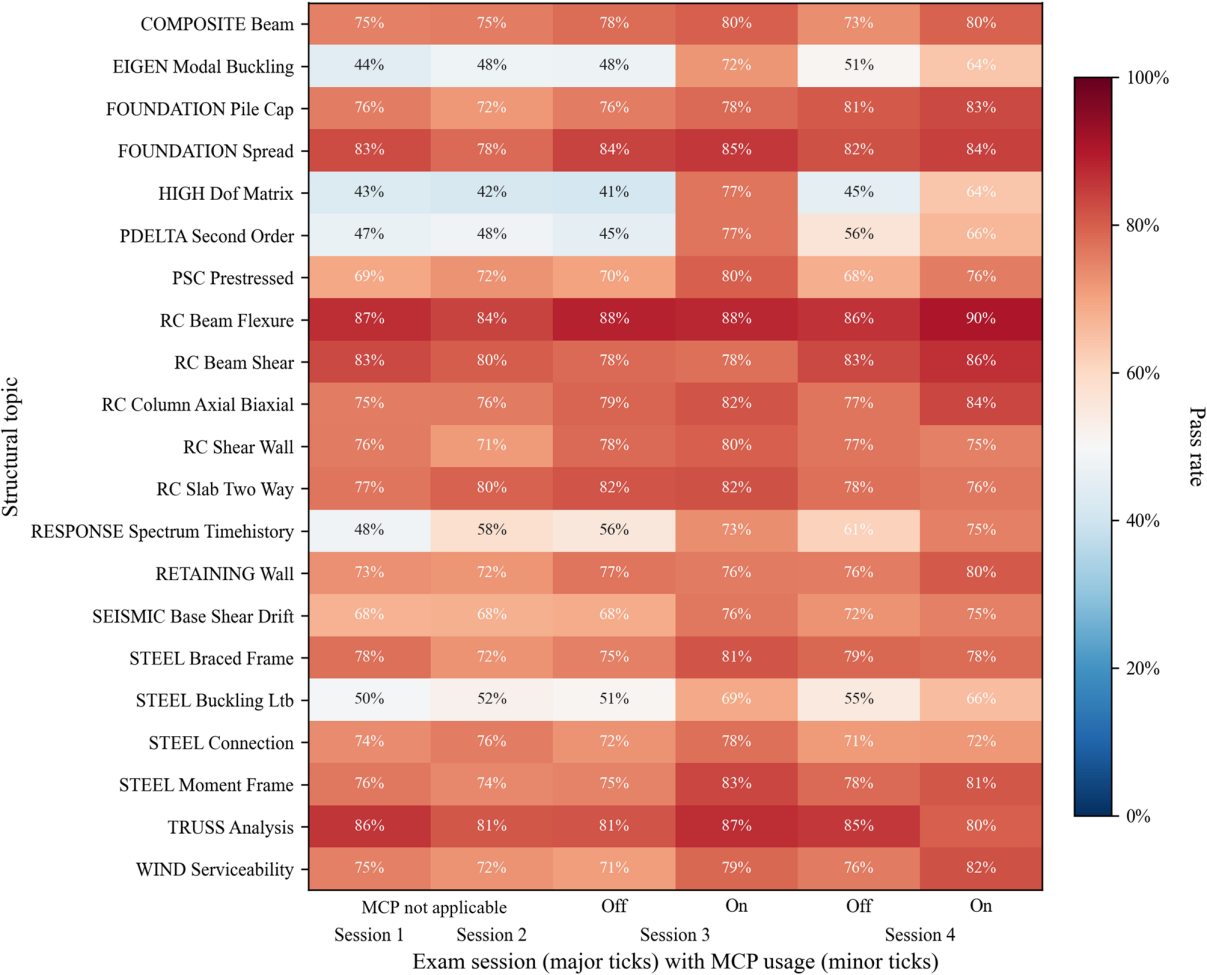
Heatmap of mean final pass rates by structural topic and exam session. Sessions 1–2 correspond to sections where MCP routing was not operationally required and are therefore shown without an MCP split, whereas Sessions 3–4 show the primary MCP Off/On comparison.

Next, the utility of the MCP hybrid approach introduced to address complex computational problems requiring numerical analysis was evaluated. Figure 12 summarizes mean final pass rates by topic and examination session. In the Korean Professional Engineer Structural Engineering examination, Sessions 1–2 are primarily descriptive or code-interpretation sections, so MCP routing is not operationally required in the intended workflow. Accordingly, Fig. 12 reports Sessions 1-2 without an MCP split. For Sessions 3–4, where numerical-analysis subtasks appear, the figure presents the primary MCP Off/On comparison under the predefined trigger policy (T1–T4: large-scale DOF, nonlinear effects, eigenvalue problems, etc.).

The results indicate that the utility of MCP routing depends on problem type and on whether the examination session actually contains numerical tasks to which the trigger policy can apply.

Sessions 1–2 (descriptive and code-oriented sections): these columns should be read as sections in which MCP routing was not operationally required; accordingly, Fig. 12 shows these sessions without an MCP split.

Session 3 (high-dimensional matrices and iterative analysis): the largest gains were observed in categories such as high-DOF matrix analysis, P-$\Delta$ second-order effects, and eigen or buckling tasks, where the predefined triggers were expected to activate more often. These results are consistent with the interpretation that policy-guided solver routing is most useful when the numerical burden exceeds a practical LLM-only regime.

Session 4 (case-based comprehensive applications): intermediate-level performance improvements were observed in applied problems such as seismic design and serviceability reviews.

Performance gains were concentrated in categories where external numerical solving was genuinely relevant, whereas lower-complexity categories changed little.

#### Analysis of context length management impact on reliability

To probe a possible mechanism behind these gains, the effect of context-length management on reliability was examined. In complex multi-stage problems like structural analysis, naive iteration methods that simply reuse all previous reasoning in the next prompt rapidly lengthen the context. This monolithic context expansion can trigger the so-called “Lost in the Middle” phenomenon, in which LLMs miss middle portions of the input and performance degrades. The proposed multi-agent pipeline was designed to mitigate that problem by keeping each agent’s context focused through stepwise decomposition and structured data (JSON, Markdown) transmission.

The context inflation ratio (CIR) is defined as the total tokens used to solve a problem divided by the baseline tokens needed for single-pass analysis without iteration. Lower CIR indicates higher token efficiency and more effective context management.

1 $$\begin{aligned} \text {Context inflation ratio (CIR)} = \frac{\text {Total tokens used}}{\text {Baseline tokens}} \end{aligned}$$

**Fig. 13:**
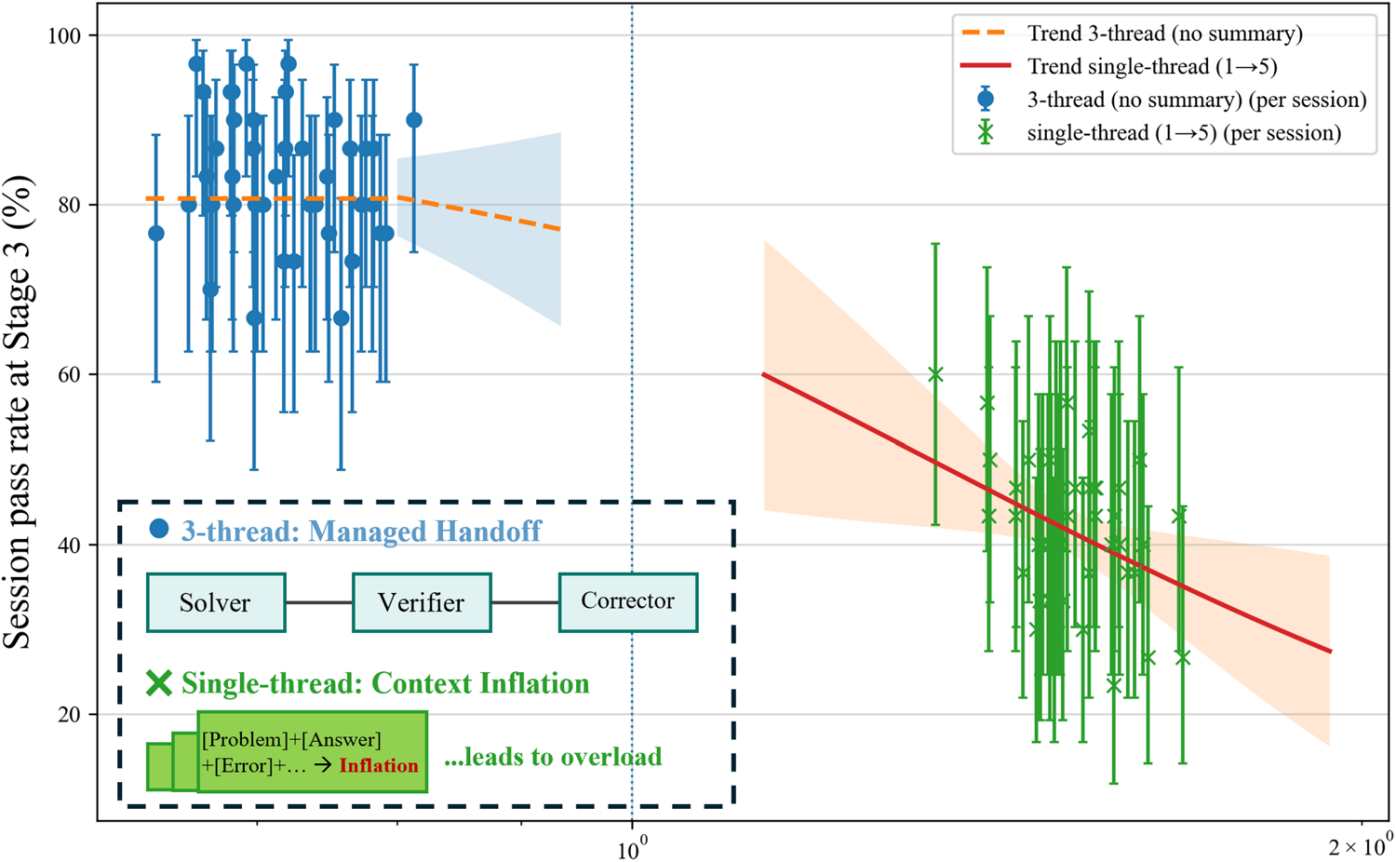
Relationship between Stage-3 session pass rate and Context Inflation Ratio (CIR); each point denotes one exam session and vertical bars show standard deviations across 10 repeated runs.

Using the repeated-run protocol described in Methods, the proposed multi-agent pipeline was compared with a baseline approach that continuously adds correction history within a single thread, expanding context. Results are shown in Fig. 13.

Figure 13 shows a clear separation between the two workflows. The single-thread baseline exhibited a negative relationship between Stage-3 session pass rate and CIR, whereas the multi-stage pipeline remained clustered in a lower-CIR, higher-pass-rate region with flatter trendlines. Figure 13 visualizes run-level standard deviations across the 10 repeated runs, and Table S2 provides a compact uncertainty summary for the same repeated-run setting using Wilson 95% confidence intervals. Across the 45 examination sessions, the multi-stage pipeline achieved a higher mean Stage-3 session pass rate than the single-thread comparison workflow (83.26% vs. 41.48%) while maintaining a lower mean CIR (0.717 vs. 1.520). The uncertainty summaries were also narrower on average for the multi-stage pipeline, with a mean Wilson 95% confidence-interval width of 25.47 percentage points compared with 32.83 percentage points for the single-thread workflow. Taken together, these results suggest that stage-wise context management was associated with lower context inflation and more stable performance in this evaluation.

## Discussion

The proposed five-stage pipeline and MCP hybrid design played complementary roles across the two evaluated cases. First, the Self-Improvement $\rightarrow$ Verifier $\rightarrow$ Correction loop improved reliability by iteratively removing defects from the initial draft rather than relying on a single-pass answer. In Case A, the loop refined the benchmark collapse interpretation by clarifying hinge candidates, plastic-capacity usage, sign conventions, and reporting consistency before the final bending moment diagram was synthesized. The Verifier stage then checked equilibrium, mechanism admissibility, and plastic-moment consistency, allowing the Correction stage to address only the flagged issues while preserving already acceptable parts of the draft. This behavior is consistent with the iteration-versus-pass-rate trend in Fig. 11, where the largest gain appears in the first loop and later gains diminish. In other words, the main benefit of the loop is not repeated paraphrasing of the same answer, but structured elimination of engineering and reporting defects through staged review.

**Fig. 14:**
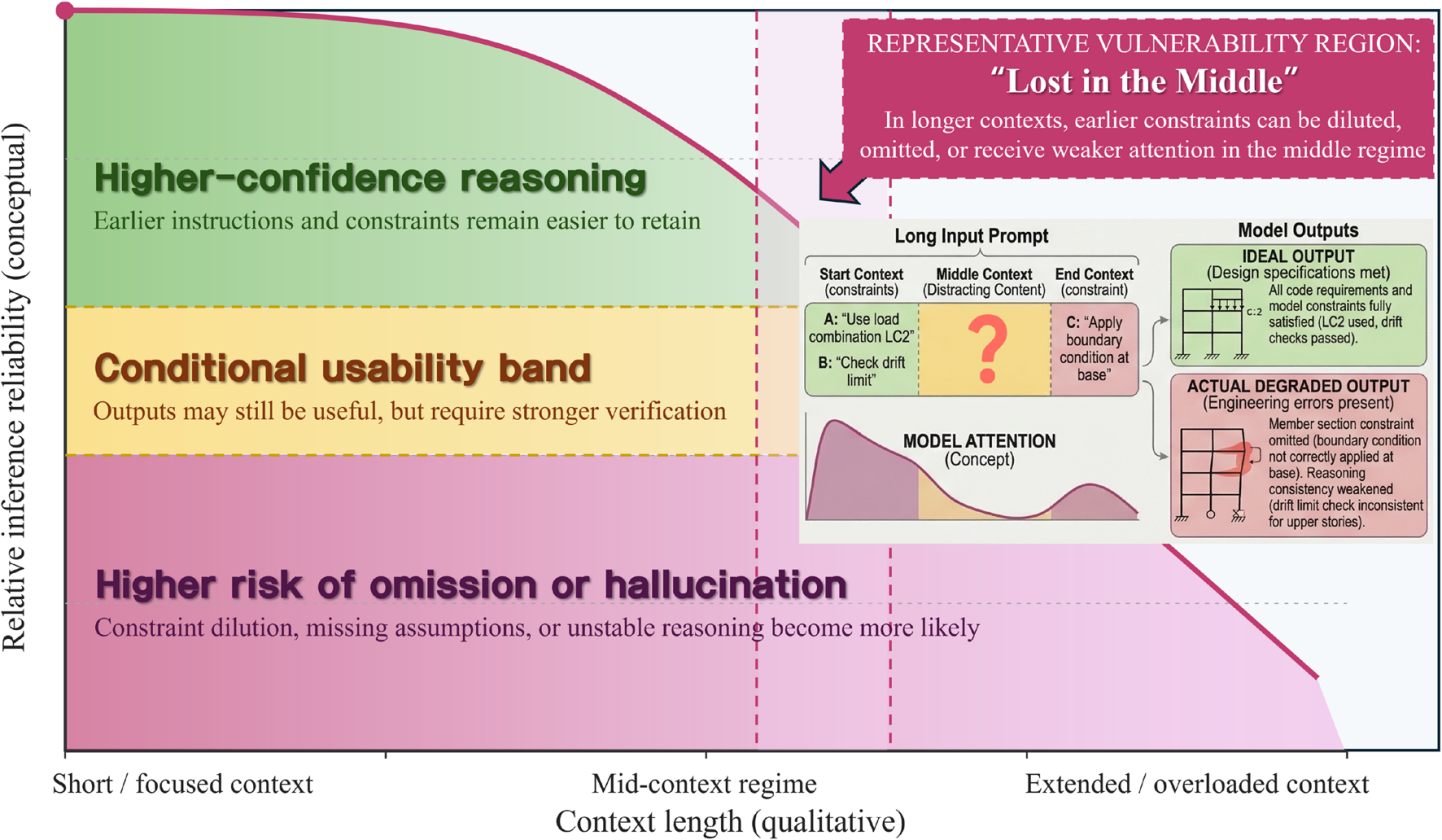
Illustrative schematic of qualitative LLM reasoning degradation with increasing context length (“lost in the middle”); this figure is not directly fitted to the experimental data in this study.

As shown in Fig. 13, the design based on stage separation and structured summary handoff also appears to reduce some of the degradation associated with uncontrolled context growth. When long structural descriptions, correction history, and intermediate calculations are injected into one monolithic prompt, important assumptions can become difficult to recover consistently in later reasoning steps. In contrast, the proposed workflow re-initializes context by stage and transmits only the necessary intermediate outputs in structured formats such as JSON or Markdown. This keeps the local reasoning scope narrower and makes the next stage’s role more explicit. Figure 14 is therefore included as an illustrative schematic of the long-context issue rather than as a fitted empirical model for this study. These trends suggest that stage-wise context management improved pass-rate stability and reduced run-to-run dispersion in this evaluation.

The MCP hybrid also improved the workflow’s efficiency-accuracy trade-off in the evaluated high-complexity case. In Case B, the T1/T2 trigger conditions routed the numerical solve to MATLAB once the problem scale and second-order requirements exceeded a practical LLM-only regime. The LLM pipeline then remained responsible for model packaging, report integration, and engineering checks on the returned result. In this configuration, the external solver handled the sparse numerical analysis, while the LLM handled interpretation, coordination, and synthesis. This role division is consistent with actual engineering practice, where large-scale linear or iterative calculations are delegated to specialized numerical engines while higher-level judgment, verification, and reporting remain separate tasks. The workflow used a general-purpose Gemini 2.5 Pro model rather than a structural-analysis-specific fine-tuned model. The result still depended strongly on how checking, correction, and routing were organized.

The gate policy results likewise suggest that verifier-driven stopping may be more practical than a fixed loop count in the present evaluation. Most gains were realized within the first one or two verification-correction loops, after which diminishing returns became apparent. However, the present experiments do not establish a validated mapping between task difficulty and optimal loop depth. Accordingly, the practical implication is a verifier-driven stopping heuristic based on consecutive Pass outcomes, absence of new Critical findings, or negligible residual changes. The Verifier’s severity labels (Critical, Gap, and Format) provide an operational cue for this stopping heuristic. The relation between task difficulty and sufficient loop depth remains a question for future work. This interpretation is also consistent with broader tool-use and pipeline-orchestration literature, where external intervention is typically most useful when triggered selectively rather than applied indiscriminately^20^.

Several limitations remain. First, the manuscript still reports only two proof-of-concept case studies, so the evidence remains exploratory and should not be read as a broad benchmark across all structural-analysis tasks. Second, problem complexity was characterized only partially; DOF was useful in Case B, but complexity in practice also depends on nonlinearity, coupling, boundary-condition richness, and reporting requirements. An additional limitation is that the present study does not establish a validated mapping between task difficulty and sufficient loop depth. Third, the current MCP implementation is specific to the tested MATLAB-based setup, and the routing policy remains predefined rather than learned adaptively from task outcomes. Fourth, the workflow remains sensitive to prompt design and inter-agent interaction rules, and the present ablation does not constitute an exhaustive study of language, template, or multilingual robustness. Fifth, the Verifier itself is imperfect, so some solver or report inconsistencies may still escape detection, which is a known limitation of LLM-based judging more broadly^5^. Finally, the current reproducibility package still depends on structured text artifacts and does not yet integrate multimodal inputs such as structural drawings or BIM-native geometry.

These limitations point to several concrete directions for future work. The benchmark suite should be expanded to include a wider range of structural-analysis tasks, solver backends, and complexity levels. Prompt robustness, resource use, and uncertainty should also be quantified more systematically across repeated runs and broader prompt variants. In practical engineering settings, tighter integration with multimodal data sources, including drawings and BIM-linked inputs, would be valuable for reducing the burden of manual problem encoding. Additional complementary verification layers may also strengthen trust in LLM-assisted structural-analysis workflows. For example, physics-based checking methods and formal verification ideas developed in adjacent reasoning fields may provide useful secondary audits of model-generated solutions^22,23^. Such extensions would make the workflow more reliable in practical engineering use.

## Conclusion

The proposed framework improved reliability and reproducibility in the evaluated structural-analysis tasks by replacing a single-pass LLM response with a five-stage pipeline of Solver, Self-Improvement, Verifier, Correction, and Synthesis. For larger numerical tasks, the MCP-based hybrid path routed selected subproblems to an external deterministic solver under predefined trigger conditions. Across the evaluated cases, this combination improved verification pass rates, produced more stable outputs than the comparison workflow, and limited uncontrolled context growth through structured JSON/Markdown handoffs.

In Case A, the verify—correct loop improved the internal coherence of the collapse-analysis solution. In Case B, the MCP path supported the larger numerical task while keeping the LLM responsible for interpretation, integration-level checks, and final synthesis. The evidence remains limited to the tested prompts, benchmarks, and routing policy. Broader benchmark coverage, stronger prompt robustness, multimodal data integration, and additional verification layers remain necessary for more dependable engineering use.

## Data Availability

All prompts, input configurations, and structured outputs that support the findings of this study are openly available at https://github.com/m-ill/Prompt-Engineer. The original Professional Engineer exam question statements and images are not redistributed due to copyright restrictions; derived metadata and evaluation scripts are provided instead. Additional data and materials are available from the author upon reasonable request.

## Code availability

Custom code and workflow artifacts used in this study are openly available in the public Prompt-Engineer repository (URL: https://github.com/m-ill/Prompt-Engineer; study snapshot used for this article: commit 7716bc751d85; accessed 18 March 2026). The repository includes prompt orchestration artifacts, input configurations, evaluation scripts, and plotting utilities used for the reported experiments.
